# When mining reshapes bodies and destinies: trafficking, gender-based violence and resilience among rural women in Kalehe and Kabare in South Kivu

**DOI:** 10.3389/fsoc.2026.1803429

**Published:** 2026-04-24

**Authors:** Chavez Cikuru Kamera, Esther Mubalama Bizuri, Jonas Nshokano Bahizire, Pépé Bwemere Kamera, Gervais Muderhwa

**Affiliations:** 1 Department of Population and Development Sciences, Université Catholique de Louvain (UCLouvain), Louvain-la-Neuve, Belgium; 2 MKAAJI MPYA asbl, Bukavu, Democratic Republic of Congo; 3 MKAAJI MPYA asbl and Angaza Institute, Bukavu, Democratic Republic of Congo

**Keywords:** artisanal mining, gender-based violence (GBV), human trafficking, structural vulnerability, women’s labour invisibilisation

## Abstract

**Introduction:**

Artisanal mining in eastern Democratic Republic of the Congo (DRC), particularly in the territories of Kabare and Kalehe in South Kivu, constitutes an ambivalent social space where economic opportunities, structural precarity and gender-based violence intersect. Drawing on an inductive, multi-sited qualitative approach, this study examines the life trajectories of rural women and girls who are directly or indirectly involved in artisanal mining economies, with the aim of analysing the social, economic and political mechanisms that produce and sustain their vulnerability to trafficking and sexual and gender-based violence (SGBV).

**Methods:**

This study adopts an inductive approach and draws on a range of data collection techniques, including semi-structured interviews, focus groups and an in-depth literature review. The study highlights a continuum between economic survival strategies and forms of exploitation, in which access to mining-related income is embedded within asymmetrical power relations, the invisibilisation of women’s labour, and institutionalised practices of domination.

**Results:**

The findings show that, far from being limited to spectacular forms of violence in contexts of armed conflict, the trafficking of women and girls is rooted in gradual, normalised, and often invisible processes linked to chronic poverty, constrained mobility, patriarchal norms, and the fragmented governance of mining sites. While mining sites offer limited spaces of economic empowerment for women, through access to independent income, increased mobility and solidarity networks these opportunities remain fragile and highly conditioned by gendered extractive structures that appropriate both women’s labour and bodies.

**Discussion:**

By uncovering the “silent cradles” of trafficking in rural mining areas, this article contributes to the literature on gender, extractivism and human security, advocating for a structural analysis of gendered violence that moves beyond humanitarian and securitised frameworks.

## Introduction

Natural resources, particularly strategic minerals, now play a central role in globalised economies, especially in a context of technological and energy transition where global demand for minerals such as cobalt, gold, coltan and lithium continues to rise (Ali et al., 2017; Calderon et al., 2020). However, while the exploitation of natural resources is often presented as a potential lever for economic development, a wealth of literature highlights that it can also lead to social marginalisation, inequality and conflict, a phenomenon often described as the resource curse (Auty, 2002; Le Billon, 2001; Sachs and Warner, 2001; Vlassenroot and Raeymaekers, 2004). In many countries, extractive economies are thus producing profound transformations in local social structures, redefining power relations, human mobility and forms of vulnerability (Bridge, 2009).

In countries in the Global South, these dynamics are particularly visible in regions rich in natural resources, where artisanal and industrial mining activities often coexist in contexts of fragile governance and marked socio-economic inequalities. For example, sub-Saharan Africa is home to a significant proportion of the world’s mineral resources, including gold, diamonds, cobalt, coltan and rare earth elements (Ericsson and Löf, 2019). In many countries on the continent, mining is largely dominated by artisanal and small-scale forms, which represent a vital source of income and livelihood for millions of people living in rural areas (Hilson and McQuilken, 2014). However, African mining economies are also characterised by high levels of informality, precarious working conditions and significant inequalities in access to resources and economic opportunities (Banchirigah, 2008). Several studies have shown that the expansion of extractive economies is frequently accompanied by various forms of violence, crime and exploitation, particularly against women (Buss et al., 2019; Bashwira and Van Der Haar, 2020; Mishra et al., 2024). Research conducted in various African countries has also shown that mining areas can become hotbeds for sexual exploitation, forced labour and trafficking, due to a combination of high economic inequality, labour mobility and weak public institutions (Hirons, 2011; Maconachie, 2011). According to Tarras-Wahlberg et al. (2017), women may be simultaneously integrated into informal mining economies and exposed to multiple forms of vulnerability, including economic insecurity, sexual violence and trafficking dynamics.

The Democratic Republic of Congo (DRC) is a particularly emblematic example of these dynamics. With an exceptionally rich mineral subsoil, notably cobalt, copper, gold, cassiterite, coltan and diamonds the DRC occupies a strategic position in the global mining economy (Geenen, 2012). However, the exploitation of these resources takes place in a historical context marked by decades of political instability, armed conflict and fragile governance. In the Great Lakes region, and more particularly in the eastern Democratic Republic of Congo, mining is a key area of analysis for understanding the complex interactions between extractive economies, armed conflict, social dynamics, power relations and gender-related vulnerabilities. Since the turn of the 2000s, a wealth of literature has highlighted the central role of the mining economy in shaping social relations, human mobility and power systems in the region (Autesserre, 2010; Bashizi and Geenen, 2014; Leonard, 2024).

In this regard, numerous studies, notably those conducted by Meger and Sachseder (2020), Banwell (2020), Turshen (2016), Meger (2016) have shown that areas rich in natural resources, particularly in the Global South, generate a rentier economy, often accompanied by an increase in crime, sexual exploitation and gender-based violence (GBV). Far from being mere spaces of economic production, the extractive economy, dominated by the production of strategic minerals such as coltan, cassiterite and gold, is accompanied by a rentier economy, the presence of armed actors, illegal marketing networks and weak state regulation (Vogel and Musamba, 2016; Iguma Wakenge et al., 2021; Wakenge, 2018). From an ecofeminist perspective, these dynamics can also be understood as the expression of a broader system of domination linking the exploitation of nature to that of women’s bodies. Ecofeminist theorists have shown that extractive capitalism tends to treat both natural resources and women’s reproductive and sexual labour as objects of appropriation and accumulation (Mies et al., 2022). According to Jenkins (2014), the expansion of extractive economies not only transforms local landscapes and livelihoods but also contributes to the commodification of women’s bodies in contexts marked by economic precariousness, militarisation and institutional weakness. From an ecofeminist and decolonial perspective, this intertwining of territorial exploitation and bodily violence is conceptualised through the notion of cuerpo-territorio, according to which violence perpetrated on extractive territories is often reflected in violence perpetrated on women’s bodies.

At the heart of these contradictory dynamics, women, particularly young rural girls, find themselves caught between two conflicting forces: on the one hand, mines offer opportunities for income and social mobility; on the other, they become spaces for sexual predation, human trafficking and exploitation, orchestrated by powerful figures in the sector. At the global South level, studies such as those by De Doris et al. (2017) on the DRC and Lahiri-Dutt (2011) on communities living around mining areas in the Global South highlight how women are both integrated into the informal circuits of the mining economy (small trade, sex work, domestic work) and exposed to serious violations of their rights.

In the specific context of the Democratic Republic of Congo, Kelly et al. (2014), Geenen and Verweijen (2017) and Geenen (2012) have extensively documented the links between instability, extractivism, militarisation and increased vulnerability of women in mining areas. While for many rural women artisanal mining is a survival strategy in an environment marked by chronic poverty, political instability, incessant wars, latent conflicts and forced population displacement, they simultaneously become breeding grounds for exacerbated patriarchal norms, male domination and more or less institutionalised systems of exploitation (Bashizi and Geenen, 2014; Kelly et al., 2014). In these contexts of significant power asymmetry, rural women and girls are often perceived as a cheap, mobile and easily exploitable labour force. The economic value of women and girls is thus exploited both by their families seeking a survival strategy and by economic actors (traders, quarry owners, merchants, military personnel) exploiting their socio-economic vulnerability (Porter, 2011).

This paradox between economic resilience and systematic endangerment poses a major challenge for international agendas on human rights, gender equality, and the protection of women and girls in fragile contexts. Although numerous NGO reports and academic studies have documented war rape and violence in conflict zones, the literature remains relatively silent on the invisible, daily and systematic forms of gender-based violence in mining contexts, as well as on the silent trajectories of trafficking in women for sexual and economic purposes. Despite this renewed interest, particularly in the wake of the Sustainable Development Goals (SDGs), especially SDGs 5, 8 and 16, respectively dedicated to gender equality; decent work and economic growth; and peace, justice and effective institutions, studies on gender-based violence in mining areas have seen a resurgence of interest. However, this research remains largely dominated by humanitarian or security approaches focused on mass sexual violence in times of war (Bartels et al., 2013; Ba and Bhopal, 2017).

This dominant focus tends to obscure the micro-social and economic processes through which mining sites become silent breeding grounds for the trafficking of women and girls. In remote rural areas such as Kabare and Kalehe, trafficking does not only take spectacular or violent forms but is part of gradual and socially normalised mechanisms promises of employment in mining centres, early marriages justified by economic security, or gradual integration into informal networks of hidden sex work.

Contrary to prevailing perceptions that reduce women’s presence on mining sites to an individual choice or voluntary prostitution, field observations show that these trajectories are deeply structured by economic and social constraints. Chronic poverty, the lack of economic alternatives in rural areas, forced displacement and the collapse of traditional livelihoods severely restrict the decision-making power of rural women and girls. This study aims to analyse the social, economic, cultural and political factors that produce and perpetuate patterns of structural vulnerability in the artisanal mining areas of Kabare and Kalehe. The trajectories observed reveal a continuum between survival strategies and exploitation, where certain situations tip over into trafficking when freedom of movement, control over income or the possibility of breaking an economic relationship are limited by third parties. By shifting the analysis from a moralistic reading to a structural approach, this contribution highlights the porosity between the informal economy, social precariousness and gender-based exploitation. She thus enriches the existing literature by showing that mining sites are not only informal workspaces, but also social mechanisms for producing dependency, control and the capture of female bodies.

With this in mind, the study has four objectives, namely: (1) to identify visible and invisible forms of trafficking in women and girls in these mining areas; (2) to analyse the socio-economic, cultural and institutional mechanisms that reinforce the vulnerability of rural women; (3) to examine the individual and collective strategies for survival, mobility or resistance developed by women in these contexts; and (4) to analyse the role of local actors (chiefdoms, traders, miners, armed groups, state authorities) in reproducing or limiting these forms of trafficking. This research therefore seeks to answer the following question: How do the life trajectories of rural women and girls in mining areas reveal the links between economic opportunities, social precariousness and vulnerability to trafficking? It is in this context that this research examines how the mining contexts of Kabare and Kalehe contribute both to opening up economic opportunities for rural women and to consolidating new forms of violence and trafficking.

### Understanding artisanal mining in the DRC

In the Democratic Republic of Congo (DRC), small-scale mining (SSM) is a key component of the national economy. The DRC has significant deposits of copper, cobalt, diamonds, gold, tin, coltan and tungsten, giving it a central place in the global mining economy which play a key role in global supply chains, particularly in the digital technology and energy transition sectors (Sovacool et al., 2020). This mineral wealth gives the DRC considerable potential for economic development, but it also comes with numerous political, social and environmental challenges. In this regard, artisanal and small-scale mining (ASM) plays an essential role in structuring the Congolese mining sector and in the livelihoods of millions of households (Hilson and McQuilken, 2014; Geenen, 2012). In certain regions of the country, particularly in the east (South Kivu, North Kivu and Maniema), artisanal mining is even one of the main local economic activities. According to Cuvelier (2014) and Bryceson and Geenen (2016), artisanal mining has become a major source of livelihood for hundreds of thousands of Congolese people, against a backdrop of transformation in the mining labour market and economic crisis. However, artisanal mining in the DRC relies mainly on manual labour characterised by manual or low-mechanised extraction and generally lacks financial and institutional support (Bikubanya and Radley, 2022).

Indeed, the expansion of artisanal mining in the DRC is part of the structural transformations that have taken place in the mining sector since the 1980s, with certain mining concessions being opened up to artisanal miners (Mazalto, 2009). The liberalisation of the Congolese mining sector and the collapse of certain public companies has encouraged the growth of artisanal mining, enabling many households to turn to mining as a means of economic survival (Makori, 2017; Rubbers, 2020). Beyond these dynamics, the armed conflicts that marked the DRC in the 1990s and 2000s contributed significantly to the disorganisation of the state, the proliferation of armed groups and the collapse of many economic structures. During this unique period, artisanal mining gradually developed as a survival strategy for many communities, who turned to small-scale mineral extraction to meet their needs (Autesserre, 2010). Despite its economic importance, artisanal mining in the DRC faces several structural challenges. On the one hand, the sector remains largely informal, which complicates regulatory mechanisms and limits the state’s ability to control mineral production and marketing chains (Geenen, 2012).

**Figure 1:**
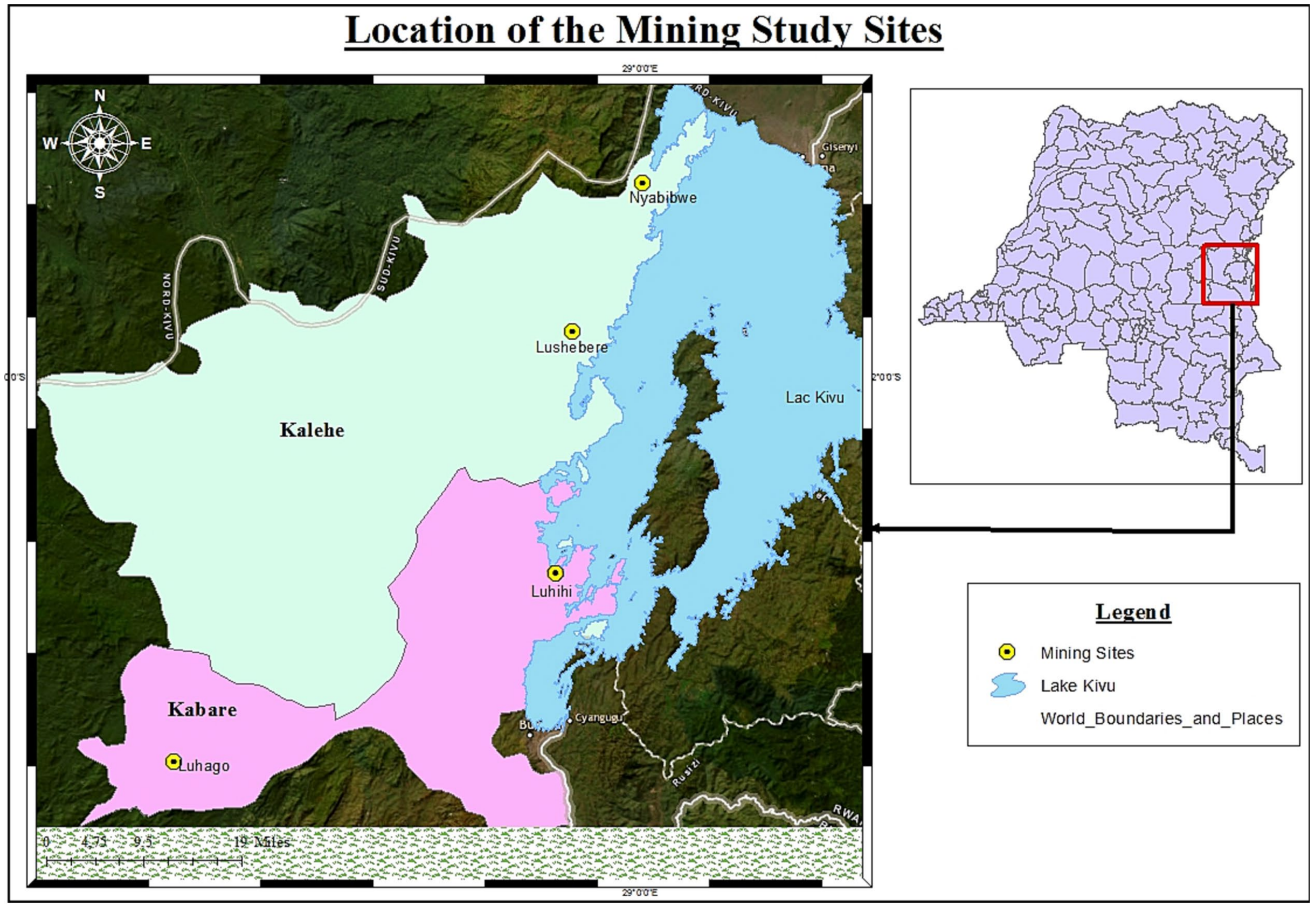
Map of artisanal mining sites studied.

On the other hand, working conditions in artisanal mining sites are often precarious, characterised by a lack of protective equipment, high exposure to accident risks and intense exploitation of labour. Artisanal mining activities are present in several regions of the country and involve different types of minerals. In the provinces of Lualaba and Haut-Katanga, artisanal mining mainly focuses on cobalt and copper (Banza Lubaba Nkulu et al., 2018). In the Kasai regions, it mainly concerns diamonds, the artisanal mining of which has historically been one of the pillars of the local economy. In other provinces such as Maniema, North Kivu, South Kivu and Ituri, artisanal mining focuses on gold and the so-called “3 T” minerals (tin, tantalum and tungsten) (Iguma Wakenge et al., 2021). This geographical and mineral diversity reflects the national scale of the phenomenon and its structuring role in the country’s regional economies (Radley and Vogel, 2015). In eastern Democratic Republic of Congo, the exploitation of natural resources is closely linked to governance dynamics and local power structures. Since the Congo wars in the late 1990s, scientific research and NGO reports have shown that mineral resources have played an important role in the political economy of armed conflict. Minerals such as gold, cassiterite, coltan and tungsten, mainly extracted from mining sites in the provinces of South and North Kivu, have been sources of funding for various armed groups as well as for certain state and parastatal actors involved in resource exploitation and marketing networks (Le Billon, 2001; Cuvelier, 2014). In these regions, artisanal mining sites are generally characterised by low-tech, low-capital extraction methods, relying mainly on the labour of artisanal miners (often organised into informal teams or cooperatives). The techniques used remain largely rudimentary: pickaxes, shovels, manually dug wells, washing and sorting of ore, and the organisation of work is based on a local economy structured around multiple actors: miners, traders, transporters, landowners, traditional authorities and mining cooperatives (Geenen, 2011; Hilson, 2016). Furthermore, this small-scale artisanal mining is deeply embedded in global supply chains through a complex system of intermediaries linking rural extraction sites to international markets (Hilson, 2016). The minerals extracted from these sites, sometimes at great cost, generally pass through local traders, purchasing houses (comptoirs) and exporters before being integrated into international industrial circuits, particularly in the electronics and technology industries Figure 1.

### Methodology and techniques

To grasp the complexity of trafficking dynamics in artisanal mining contexts, this study adopts an inductive approach and a multi-situated qualitative methodology, focusing on individual experiences, narratives and trajectories, while employing an intersectional and contextualised approach to gender, rurality, precariousness and mobility. The choice of an inductive approach is justified by the complexity of the phenomenon we are attempting to study: trafficking dynamics, which are largely informal, are intertwined with local socio-economic networks, asymmetrical gender relations and fragmented governance practices. An open, exploratory approach rooted in lived experiences was therefore necessary to grasp the underlying logic that eludes strictly theoretical analyses Table 1.

**Table 1:** Typology of actors encountered at artisanal mining sites.

Category of actors	Specific actors	Main roles/Functions
1. Mining production stakeholders	Diggers (artisanal miners)	Ore extraction, excavation, internal transport.
	Carriers/transporters	Transportation of ore to processing points.
	Underage women (crushing, sorting, washing)	Primary ore processing tasks.
2. Economic and commercial actors	Traders/trading posts	Purchase and sale of ore; informal price fixing.
	Commercial intermediaries	Local resale chain between miners and trading posts.
	Mine foremen/team leaders	Financing, work organisation, team supervision.
	Informal traders	Sale of food, equipment, drinks, small services.
	Industrial buyers	Procurement from trading posts or through formal channels.
3. Institutional players	SAEMAPE, Mining Division	Technical supervision, legal taxation, control.
	Mining police/FARDC/ANR	Security, access control, sometimes informal taxation.
	Local administrations (group leaders, villages)	Local governance, customary authorisations and regulation.
4. Customary and community actors	Traditional authorities	Traditional land management, conflict mediation.
	Mining cooperatives	Organisation of miners, administrative supervision.
	Riverside communities	Population affected by mining activities.
5. Non-state actors	Local and international NGOs	Human rights protection, gender programmes, compliance monitoring.
	Armed groups or militias	Illegal taxation, parallel security checks.
	Civil society, journalists	Monitoring, advocacy, reporting abuses.
6. Vulnerable actors	Women and girls in precarious situations	At-risk workforce, exposure to GBV and exploitation.
	Children in mines	Hazardous work and economic exploitation.

This research is a continuation and extension of an initial baseline study we conducted as part of the project for the socio-economic empowerment of women and girls with a view to combating sexual and gender-based violence (VSBG) in the territories of Kalehe and Kabare, through MKAAJI MPYA asbl in collaboration with The Circle, which documented informal forms of exploitation, invisible violence, the working conditions of women and girls in the mining sector, and precarious female migration in 12 rural localities around artisanal mining sites. The data from this initial exploratory phase, based on exploratory interviews and community discussions, provided the empirical and methodological basis for this study. The current aim of this article is to enrich, expand upon and cross-reference this data through a strengthened methodological approach. In this regard, the work carried out with the Rural Women’s Leadership Centres (RWLCs), established as part of the aforementioned project, has been a key factor in enriching and consolidating the data. Furthermore, the experiences shared during training sessions, workshops and discussion forums have significantly contributed to deepening and triangulating the findings from the interviews. With regard to the study area, research was carried out in four key mining corridors: Mudaka-Cibanda (Kalehe & Kabare), Tchulwe-Irhegabarhonyi-Nindja (Kabare), Minova-Nyabibwe, and Bushushu (Kalehe). These areas have contrasting yet similar socio-economic, mining and security characteristics, while sharing a number of underlying dynamics: the presence of artisanal mines, rural poverty, the militarisation of mining sites, informal mineral exploitation networks and a growing number of women and girls engaged in economic activities in the surrounding areas.

In order to gather different perspectives and understand the power dynamics at play, several categories of actors were targeted: First, 40 semi-structured interviews and 10 focus groups were conducted with rural women and girls aged 15 to 45 who had experienced or were constantly exposed to some form of forced mobility, sexual exploitation, transactional sex, or precarious domestic work around an artisanal mining site in Kalehe and Kabare specifically. Women in this age group work directly or indirectly in the mining economy, which enabled us to understand individual trajectories, asymmetrical power relations, survival strategies and experiences of domination. Secondly, 35 semi-structured interviews and 10 focus groups were conducted with actors in the mining sector (artisanal miners, traders, mining cooperatives) in order to understand male perceptions of women’s and girls’ work in mining sites, authority and control of natural resources, and to analyse the reconfiguration of male power relations induced by the extractive economy. The mining industry stakeholders were approached using the snowball technique. Initially, three industry stakeholders were approached individually for interviews, and we asked them to recommend others for the same exercise. Overall, the interviews lasted between 50 min and 1 h 20 min, depending on the questions and experiences that each person had to share with us as part of this research.

In addition, 15 focus groups were conducted with community leaders, traditional chiefs and local NGOs defending human rights and minority rights, who observe these dynamics on a daily basis and interact with all the socio-professional and socio-economic categories mentioned above. Finally, invisible intermediaries (mama-colas, informal recruiters of women workers in mining sites, transporters, mining operators, etc.) who participate directly or indirectly in trafficking and artisanal mining circuits. In addition to these data collection techniques, we also used documentary research, which allowed us to analyse existing texts such as NGO reports, mining policies, legal texts, and academic studies on gender and natural resources. This technique, in the context of this study, allowed us to contextualise the phenomenon, understand its complexity, and identify different analytical frameworks. Here is a comprehensive list of the stakeholders we met during this study.

Given the complexity of the issues addressed (in particular: combating sexual and gender-based violence in mining communities, prevention and awareness-raising activities, the socio-economic empowerment of survivors, local leadership and governance, as well as livelihoods linked to agricultural activities), particularly those relating to women’s experiences in mining areas, it is worth highlighting the importance of the support provided by pre-existing local structures, established as part of the aforementioned project. Among these, the RWLCs (Rural Women Leadership Centres) of MKAAJI MPYA asbl provided a key point of entry. They not only facilitated access to the field but also helped to enhance the quality of the information gathered, thanks to their strong community roots and in-depth knowledge of local realities.

These structures, established in several villages across the study areas, played a decisive role in establishing links with stakeholders directly or indirectly involved in mining dynamics and women’s economic activities. They thus enabled us to move beyond a superficial reading of the data to gain access to lived realities, situated narratives and deeply contextualised experiences.

Beyond these institutional and community dynamics, our role as stakeholders involved in the implementation of the project led served as a key methodological asset. Indeed, our experience with NGO-led projects in the Kivu region, amongst others, in the implementation of development projects and programmes, has facilitated a more nuanced understanding of the contexts under study. This experience also enabled us to strategically identify the relevant categories of stakeholders to be mobilised, whether they were the women directly affected, community leaders, mining operators, local authorities or civil society organisations.

This table presents the main categories of actors involved in artisanal mining sites, their position in the chain of activity, and their respective roles within local economic, social, and security dynamics. These actors, each in their own role and with complementary information, enabled us to document our study on the vulnerability of women in and around artisanal mining sites in the context of South Kivu province. The interviews were conducted in three languages: Firstly, in Kihavu and Mashi (vernacular languages spoken in the territories of Kalehe and Kabare) for the majority of the people approached, particularly women and girls; in Swahili for other people from different categories; and finally, in French, generally with local authorities and representatives of local structures such as human rights and minority rights NGOs. These interviews were transcribed in their original languages for analysis in English.

In order to analyse the data collected using the various techniques and methods described above, we conducted a thematic analysis using an inductive coding approach. This approach enabled us to code the data from individual interviews and focus group in order to identify recurring themes such as empowerment, marginalisation, resilience and the gender division of labour. These themes were then grouped into broader analytical categories, including socio-economic barriers, survival strategies and gender dynamics. This structure enabled us to interpret the participants’ statements by highlighting the social logic that shapes their participation in artisanal mining activities. From a comparative perspective, the results were also analysed across the different sites studied (Nyabibwe, Lushebere, Luhihi and Luhago) in order to identify local variations and understand how geographical, social and institutional contexts influence the opportunities and constraints that shape women’s participation in these mining areas. These results can be consulted below in the section devoted to the presentation of results.

## Results

### Role of mines as a source of income and potential spaces for empowerment for women and girls

For women and girls, work around artisanal mining sites plays a central role in the local economy and represents an essential source of income for many families. In these areas, where structural poverty, scarce employment opportunities and limited access to productive land have a significant impact on households, mining becomes one of the few places where it is possible to earn a regular, albeit modest, income. For many women, working at mining sites is not a choice, but a survival strategy aimed at ensuring their families’ livelihoods. They are directly involved in the artisanal mining process through activities such as crushing, washing, sieving and sorting minerals. Although these tasks are physically demanding and poorly paid, they remain essential to the production chain. Despite their fundamental contribution, their work is often considered secondary and remains largely invisible in official statistics and mining policies. However, this economic centrality contrasts sharply with the precariousness of working conditions and remuneration. Despite their remarkable work, their remuneration generally depends on the quantity processed or the generosity of the operators, which exposes women to great economic instability. At the same time, a large number of women are involved in related economic activities around mining sites. They sell food, drinks, coal, clothing and other essential items. Some run small makeshift kiosks or provide services such as cooking and laundry for miners. These micro-activities, although modest, often provide a more regular source of income than working directly with the ore and allow women to maintain a certain degree of financial independence. Girls, meanwhile, sometimes go to the mines to finance their basic needs, such as schooling or helping their families, which exposes them to particular risks. However, this diversification of economic strategies does not mean an end to vulnerability.

The mine therefore represents an ambivalent space for women and girls: a place of economic opportunity, but also an environment marked by precariousness, lack of social protection, low wages and unfavourable power relations. Their real contribution to the mining economy, although decisive, is little recognised, and their presence remains marked by persistent inequalities. Thus, even though mines provide a vital source of income, they also perpetuate a cycle of economic and social vulnerability for women and girls in Kalehe and Kabare. However, mining sites, despite their precarious and often dangerous nature, offer women and girls certain spaces for potential empowerment. These spaces remain limited, but they provide crucial opportunities to access a form of economic and social independence in contexts where almost all opportunities are accessible to men. Today, for example, mines allow women to generate their own income, independent of the control of their spouse or extended family. Activities such as small-scale trade in food, beverages and charcoal, or services such as cooking, washing minerals and light mineral transport, provide a daily income. Even if modest, this income gives women some leeway in managing household expenses, purchasing personal goods, or participating in certain family decisions. This financial autonomy, however invisible and partial, can strengthen their ability to negotiate, protect themselves, or support their children’s education. As a 34-year-old woman who runs a catering business near a mining site in Luhihi said, “Here in the mine, it’s not easy, but at least I can earn something for myself. Every day, I prepare foufou and beans, which I sell to the miners. With this money, I can buy soap, pay for my children’s schoolbooks and no longer depend entirely on my husband. In the village, they used to say that a woman shouldn’t move around too much, but here I can move freely and meet other women who are experiencing the same difficulties. We help each other a lot, and that gives me the strength to carry on despite the risks”.

Secondly, mining sites create opportunities for mobility. Unlike in their home villages, where women’s mobility is sometimes restricted by social norms, women and girls who frequent the mines move more freely between different sites, local markets and villages. This work-related mobility broadens their access to information, economic opportunities and new social or professional networks. Some of the women we interviewed told us that this mobility allows them to circumvent certain patriarchal constraints in the domestic sphere on a daily basis. The interviewees stated that the presence of women in the mines facilitates the creation of solidarity and mutual aid networks such as AVECs (village savings and credit associations) and tontines, which provide them with regular assistance in the event of illness, childbirth or accidents. Generally speaking, they come together to work, secure sales areas, share risks, exchange strategies, or provide financial assistance to one another. These informal networks play a key role in strengthening their resilience and bargaining power with operators, buyers, and local authorities. Thus, despite persistent vulnerabilities, the mines in Kabare and Kalehe offer opportunities for empowerment which, when strengthened, can improve the socio-economic position of women in these territories.

### Invisibility of women’s work and gendered division of labour in mining sites

Regarding social constructs surrounding women’s work and presence in mining sites, numerous studies have shown the importance of all family members’ contributions to household income, particularly that of women. As some researchers have pointed out, women’s work in this sector often remains irregular, invisible and difficult to quantify, even though it is an essential resource for household survival (Lahiri-Dutt, 2011, Hilson et al., 2018). Studies conducted by several researchers have highlighted significant disparities in women’s participation in artisanal mining (Kamundala, 2025; Maclin et al., 2017; Bashwira et al., 2024). Despite some progress in the gender division of labour, social norms, beliefs and patriarchal values continue to limit women’s full participation in this sector. These barriers prevent women from reaping the full benefits of their labour, even though the income generated could significantly contribute to the economic and social well-being of their households. It should be noted that women play a decisive role in mining throughout the Democratic Republic of Congo, although their tasks differ considerably from those of men. Their contribution is mainly in processing, logistical support and local economic activities around mining sites. Often unaware of their rights regarding access to and control of mineral resources, they find themselves confined to low-paid activities such as crushing, washing, sieving and sorting minerals. This behind-the-scenes work, which is nevertheless essential to the extraction process, remains undervalued and places them in a situation of economic marginalisation. Furthermore, beyond productive tasks, the presence of women in the mining sites of the Kabare and Kalehe territories is also accompanied by increased forms of exploitation and vulnerability.

Those interviewed revealed cases of sexual exploitation, of which they are victims at the hands of certain operators and mineral owners. As a woman engaged in small-scale trading around a mining site in Kalehe explained, “Being a woman here means being constantly exposed. Some operators believe that our bodies are part of the price we have to pay for access to work or minerals. We suffer this violence in silence because we depend on these places to feed our families.” This reality illustrates the persistence of structural inequalities where access to mineral resources is not only gendered, but also shaped by power relations, dependency and violence. Indeed, women, often constrained by poverty and a lack of economic alternatives, find themselves in positions of subordination that expose them to physical abuse (beatings, sexual assault, rape), psychological abuse (humiliation, blackmail, threats) and economic abuse (extortion of income, non-payment for work done, conditional access to mining sites or minerals). These dynamics reinforce a vicious circle: while they provide essential labour for mining activities, they remain deprived of social recognition, economic autonomy and equitable access to the benefits of exploitation. In some cases, this precariousness even pushes some women to resort to sex work as a survival strategy, which increases their vulnerability to sexually transmitted infections (STIs), early pregnancy and widespread social stigmatisation. In addition to these factors, interviewees noted that, in such situations, the lack of adequate social and legal protection mechanisms, combined with the low representation of women in mining cooperatives or local decision-making structures, limits the ability of women and girls to assert their rights and negotiate. In this regard, a 23-year-old respondent involved in the transport of minerals states “Here, in the mining sites, when a woman is the victim of injustice, there are almost no mechanisms to protect her. The cooperatives are run by men, and we women still have no place in them. As we are not represented, we cannot negotiate our working conditions or defend our rights. Even when we speak up, our voices do not count.” Customary practices and patriarchal norms thus continue to be a major barrier to their empowerment, perpetuating unequal relations in terms of access to, control and management of mining resources.

### Challenges faced by women and mechanisms for resilience in accessing mines

The majority of women and girls in the territories of Kalehe and Kabare in particular, and in the province of South Kivu more generally, face numerous obstacles in their attempts to access mining activities. These difficulties are part of a highly gendered context, where mining remains socially and historically dominated by men. However, in the face of these structural constraints, women are also developing resilience strategies such as: 1) diversifying income-generating activities (small-scale trade, artisanal processing, daily labour), 2) resorting to subsistence farming on small plots of land, 3) participating in community savings and credit associations; in order to integrate, as best they can, into this hostile environment. However, this resilience manifests itself differently depending on the area of activity, particularly in mining sites where gender relations take specific forms. At some mining sites, notably Nyabibwe, persistent narratives attributing the disappearance of mineral veins to women continue to be mobilised by men. These beliefs contribute to maintaining negative perceptions of women, who are seen as sources of vulnerability or danger to mining activity, and are often used to legitimise their exclusion. However, despite these socio-cultural barriers, women are gradually managing to create spaces for themselves in the mining sector. Some have succeeded in establishing themselves as mineral traders, a position considered relatively high in the local social and economic organisation, particularly at the Nyabibwe site. A female leader, aged between 30 and 45, who works at the Luhago mine site, says: “When we arrive at the site, some men say that our presence causes the veins to disappear. Because of these words, we are prevented from entering certain mines or treated as bad luck”.

Several men working at various mining sites in Kalehe and Kabare claim that during menstruation, women are less able to work effectively and obtain the necessary resources. They sometimes associate this with a time when fatal accidents are more frequent in the quarries. This belief, although subjective, is dangerous because it helps to justify the exclusion of women from mining work and perpetuates discriminatory social practices that restrict their full participation. However, these obstacles do not completely discourage women; they continue to develop strategies to circumvent these restrictive norms and assert their place in the local mining economy. “Even though they say women shouldn’t go down into the mines, we have found other ways to exist in this sector. Some of us have become traders or intermediaries, because that was the only door left open. It’s not a privilege, but a strategy to circumvent prejudice and ensure the survival of our families”. In line with this approach to resilience, many women from surrounding villages are members of village savings and credit associations (VSCA) and many other solidarity structures set up by themselves, as a way of demonstrating their leadership and capacity for innovation. These structures enable them to contribute to meeting their basic needs, supporting their families and financing small projects such as agriculture or retail trade. Although these associations are not yet involved in large-scale development projects, they play a crucial role as an economic safety net and in promoting solidarity among women. In the mining sites of Kalehe and Kabare, there are also women known locally as « Shahouleurs^1^ » in Kihavu and Mashi, two vernacular languages in South Kivu province. They form associations whose aim is to clean the material and facilitate the purchase of sand. These women play a central role in the process of extracting and marketing mineral resources: they carry out processing, sorting and logistics, thus ensuring that the minerals reach the market. A woman known as “Shahouleure,” aged 29 and involved in the mining industry for the past three years, explains:

“My husband and I work in the quarry. We don’t have much money. He is a porter and I am a Shahouleur. It’s very hard work, but we have no other choice. This work is really complicated because sometimes you go to clean sand and you find that it’s good work, other times I lose out and my husband can’t understand that, even though we can’t earn money every day. It’s a big, stressful job. Sometimes my husband gets jealous because we buy sand from men and we have to beg them to sell it to us at a good price. Sometimes they flirt with you and you have to be strong, and when it gets annoying, you have to change customers, but even the new ones will end up flirting with you too, and you have to change again. Sometimes you end up with the one who has already courted you, so you have to send a colleague who will not always be transparent with you or who is not as lucky as you in choosing the sand. Many Shahouleurs fall like that. Many households collapse in this way”.

Furthermore, these mechanisms of organisation and solidarity demonstrate that, despite the constraints, women are not merely passive actors: they negotiate, innovate and adapt to the difficult conditions of mining sites. Their presence, although often underestimated, is a crucial lever for local economic development and a space for progressive empowerment, revealing a capacity for initiative and resilience that challenges traditional social norms. Most Shahoulers are women living alone, often because their spouses have left home or are absent due to work commitments far from their families. More specifically, these women bear full responsibility for the survival and well-being of their children, having to provide for their education, housing, food and safety. Their role goes far beyond simple mining activities; it is part of a complex system of family survival, where they must juggle domestic tasks, childcare and work in the mines. Alongside the “Shahouleurs” are the women porters, whose role is to transport sand and stones extracted from the mines to storage areas, homes or points of sale. They also help to transport raw minerals that can be crushed to facilitate extraction. These women often work in extremely difficult conditions, including heavy loads, unstable terrain, exposure to dust and noise from machinery, with little or no physical or social protection measures. To protect their work and their income, they must try to organise themselves into collective associations, but these are often poorly structured and lack resources or institutional support, limiting their ability to achieve their goals and improve their conditions. Despite these difficulties, these groups are a real mechanism for resilience, enabling them to support each other, exchange advice, share market information and create the beginnings of collective economic security. A woman carrying minerals extracted directly from the cassiterite pit talks about her situation:

“I am a porter in the quarry. If I had another choice, I would not do this job. My husband does nothing and has nothing. We have children to feed and clothe, and they are studying because education is free. I have already had one abortion because of this job. We carry loads heavier than our own weight. Sometimes the customers cheat us; they give you a 40kg parcel and tell you it’s 30kg. We travel long distances and it exhausts us. When you get home, you’re unable to fulfil your marital duties, your husband doesn’t understand and thinks it’s become a habit. Sometimes he hits you, insults you, calls you all sorts of names, sometimes he accuses you of having other men, and some men end up leaving or marrying another woman. Mine is still here, maybe because he has nothing else”.

The interviewees stated that women who carry minerals and mostly live alone are very often forced into this choice by the economic and social circumstances they face. Their daily lives are dominated by transporting minerals and sand, a physically exhausting, poorly paid and little-recognised activity, which is nevertheless the main means of supporting their families. Most of the women interviewed said that they combine this work with running their households in order to ensure that their children attend school, prepare meals and maintain a shelter, all in precarious conditions and with constant exposure to the risks associated with mining. In addition, there are also women in Nyabibwe who engage in commercial sex work to meet the growing demand from miners and various mining operators present at the sites. This group of women is mainly concentrated around the mining sites, seeking to earn an income from the massive influx of miners and migrant workers. In Luhihi, in the Kabare territory, these women can also be found both on the mining sites and in their immediate surroundings, forming a network of informal activities that responds to the economic demand generated by mining. Among them are women who have left other rural areas in search of better economic opportunities. One of them, a young woman who has been working in a local bar for about a year and a half, recounts her experience: “I run a refreshment bar. Customers meet us here because we can’t access the quarry, which is the main concern in our work. Once inside, we know who got what and who didn’t, so we can prepare our attack. I’m not from here. I come from far away, so I came here to make money, and nothing but money. So, we wait for the men outside”.

### Social, economic and security vulnerabilities related to women in mining sites

For several respondents, in a context of unequal access to natural resources, such as agricultural land and, more importantly, mineral resources (gold, cassiterite, coltan and wolframite), women and girls are constantly confronted with profound social, economic and security vulnerabilities closely linked to precarious living conditions, power relations and the absence of institutional protection mechanisms. Indeed, they often have limited access to extraction sites and the economic benefits they generate. During the interviews, respondents suggested that reducing inequalities in access to mining resources, particularly for women, could promote more inclusive participation in the economic benefits generated by the extractive sector and thus contribute to strengthening their livelihoods. The dynamics observed in the mining areas of Kabare and Kalehe show that women are particularly affected by the combined effects of these factors, reinforcing their marginalisation. These dynamics of marginalisation are expressed in different ways in several areas, starting with the social sphere. According to a woman, believed to be between 20 and 30 years old, who has been running a small business for the past year at the Luhihi site in the Kabare district, “Life is very hard for women at the mine site. We work in gruelling conditions, often without safety measures, and we are exposed to various forms of violence. As there are no protection mechanisms in place, we often have to fend for ourselves”.

According to the people we met, mining sites create opportunities for mobility, meaning that some women are economic migrants. They mainly come from surrounding rural areas, particularly farming villages where employment and income-generating opportunities are limited. Several factors explain this migration. On the one hand, there is economic insecurity in the villages of origin, marked by low agricultural incomes, land conflicts, food insecurity and a lack of economic opportunities for women. On the other hand, there is the economic attractiveness of mining sites, which are perceived as places where it is possible to find or quickly develop income-generating activities, often informal ones. Upon arrival at the mining sites, women engage in various economic activities related to the mining economy (washing minerals, transporting minerals, selling drinks, etc.). However, due to precarious socio-economic conditions and a lack of social protection, some women may also find themselves involved in high-risk activities, including sexual exploitation or survival prostitution (sexual transactions: exchanging sex for money or minerals). As a result, some women migrate alone, while others join their spouses or partners already engaged in mining activities out of a need to live with their families and partners.

Thus, on a social level, patriarchal norms are one of the main determinants of gender inequality. These norms assign women subordinate roles (e.g., domestic and reproductive responsibilities: childcare, housework, meal preparation, considered a priority over productive activities), both in the community and in the productive sphere of mining, where their contribution is generally perceived as secondary, informal or ‘ancillary’. This systemic devaluation, deeply rooted in local social structures, has a direct impact on women’s place in community interactions and their ability to access resources or economic opportunities. “We are there every day, but it’s as if we are invisible. When people talk about work in the mines, they always talk about men, never women”. Women’s work in the mines, limited to tasks considered less technical or less “noble,” becomes socially invisible, which contributes to reinforcing their dependence and reducing their bargaining power.

Stigmatisation is another key aspect of these social vulnerabilities. Women and girls working in mining sites are often associated with behaviour considered ‘immoral’ or with excessive social and geographical mobility. This socially constructed perception leads to processes of exclusion, ranging from social isolation to loss of status within the community. For some of the women interviewed, this stigmatisation can result in a break with their family of origin, increasing their vulnerability and exposure to other forms of exploitation. Thus, the combination of gender norms, social marginalisation and lack of institutional protection creates a structural framework that perpetuates the exclusion of women and limits their ability to participate fully in the management and equitable exploitation of natural resources. With regard to economic vulnerabilities, the people interviewed stated that women and girls mainly perform low-paid tasks: crushing, washing, sorting, small-scale trading and informal catering. Their income is heavily dependent on operators, miners or fluctuations in the mining market, which places them in a situation of chronic economic instability. This division of labour reflects not only restrictive social norms, but also an economic system that places little value on jobs considered to be feminine.

Women’s economic dependence on mining operators, traders or miners appears to be a determining factor in their precarious situation, as women would also like to mine using their own physical strength. However, the income they generate is extremely volatile and subject to seasonal variations and the monopoly logic imposed by the actors dominating the commercial circuits. The almost total absence of savings schemes, start-up capital or access to credit prevents women in particular, but also a larger number of actors in the mining sector, from pursuing alternative activities or embarking on robust paths to economic independence. The growing presence of very young girls in mining activities reflects the deep-rooted nature of this economic vulnerability in family and community dynamics. Entering mining sites due to a lack of educational or professional opportunities, these adult or adolescent girls become an exploitable workforce, often without legal protection or recognition of their rights. Their early entry into the mining sector increases the risks of exploitation, both economic and societal, and considerably reduces their prospects. Thus, the economic vulnerabilities observed are not the result of individual choices, but are part of an unequal production system, devoid of social protection mechanisms and dominated by rent-seeking behaviour. In addition to these social and economic vulnerabilities, there are closely related security vulnerabilities. The security vulnerabilities observed in the mining areas of Kabare and Kalehe are part of a structural context marked by fragile local governance and a highly intermittent state presence. This security instability does not only reflect institutional weakness; it also encourages the emergence and influence of armed actors, informal power networks and economic interest groups around the mines, who impose their own rules and modes of control.

In this unstable environment, women and girls are the most vulnerable group, both because of their often-marginalised socio-economic position and the persistence of deeply unequal gender relations. In this context, women find themselves at the intersection of socio-economic, security and symbolic vulnerabilities. Field data indicate that harassment, physical assault, threats and sexual violence are not isolated incidents, but part of structural dynamics of exploitation. Access to mining resources, employment opportunities, mobility and marketing channels is often conditioned by deeply asymmetrical power relations, placing women and girls in situations where they are forced to accept sexual exchanges under economic pressure. This logic is sometimes explicitly acknowledged by certain dominant actors in the sector, as illustrated by this statement collected during an interview: “On site, we decide who works and who sells. Those who do not accept the rules have no business here”. These power relations are reinforced by the concentration of power in the hands of mine bosses and informal authority figures. In short, the absence of institutional mechanisms for complaints and recourse, combined with fear of reprisals and social stigmatisation, contributes to maintaining a climate of structural impunity. This institutionalised silence helps to normalise abuses and limits any attempts at psychosocial or legal support.

### Role of local actors in perpetuating or limiting trafficking and gender-based violence in and around mining sites

The results of the study show that the dynamics of trafficking in women and girls in and around the mining sites of Kabare and Kalehe are part of a complex system of social interactions, characterised by a high degree of ambivalence among local actors. In these areas marked by the expansion of the extractive economy, chiefdoms, traders, miners, armed groups and state authorities participate, directly or indirectly, either in the reproduction of mechanisms of structural vulnerability or in their limitation through certain forms of social regulation and local governance. Interviews with women and girls living around mining sites highlight that traditional authorities occupy a central position in local social organisation. However, their capacity for intervention is often constrained by the economic dynamics and power relations generated by mining activity. As one woman interviewed in a village near a mining site in Kabare explains: “The chiefs know what is happening around the mines. They see the girls who come to work in bars or restaurants. But they often say they cannot prevent this because the entire economy of the village now depends on the mines”. Another participant we met in Kalehe also highlighted this institutional ambivalence: “When a situation becomes serious or when there are conflicts, the chiefs can intervene. But a lot of things happen in silence, and no one talks about them openly.”

Furthermore, local traders and economic actors appear to be key players in structuring the informal economy around mining sites. For many rural women, catering, small-scale trading and beverage sales are economic survival strategies in a context marked by precariousness and a lack of employment opportunities. However, interviews conducted at mining sites reveal that these activities can also expose some women to more or less invisible forms of economic and sexual exploitation. A young woman in her twenties, interviewed at the Luhihi mining site, explains: “I came here initially to work in catering, but sometimes the earnings are not enough, and some girls start accepting other offers in order to survive”. Another participant in Luhago indicated that these dynamics are closely linked to the socio-economic constraints faced by rural women: “Many girls come here because there is no work in the villages. They think they will find opportunities in the mines, but sometimes this exposes them to several risks.” Furthermore, a woman involved in various activities, including buying minerals and running a small business, also states that: “Even when girls are subjected to violence, they are reluctant to report it. Some say that local authorities, the police or other officials do not take their complaints seriously. Without money or support, they do not know where to seek justice. That is why many continue to work in these difficult conditions, despite the abuse and fear.”

In these extractive areas, characterised by a high concentration of male workers and rapid income circulation, mining sites also become places where gender relations and women’s livelihood strategies are reshaped. The presence of miners creates significant demand for informal services, helping to attract many women and girls in search of economic opportunities. However, this economic mobility can also reinforce dynamics of economic dependence and situations of exploitation. At some sites, the presence of armed groups or state security actors also contributes to a climate of insecurity and domination, accentuating the vulnerability of women and girls in these mining areas. Participants describe power relations marked by fear and coercion, which can encourage certain forms of exploitation. A woman at the Nyabibwe site, aged around thirty and with work experience in various fields at different mining sites in South Kivu, stated that: “When the armed men arrive on site, everyone is afraid. Some girls may agree to have sex with the leader to avoid problems or simply to be able to continue working”. These testimonies illustrate how security dynamics and power asymmetries can increase the risks of exploitation and reinforce mechanisms of domination in mining areas.

Finally, local state authorities are theoretically responsible for protecting populations from trafficking and gender-based violence. However, the study’s findings indicate that their capacity to intervene often remains limited in these peripheral areas, due in particular to a lack of institutional resources, weak administrative presence and the complex socio-economic dynamics specific to artisanal mining economies. Several participants expressed a sense of institutional abandonment and weak state protection. As explained by a woman in her 40s or 50s interviewed in Kabare, who is involved both in women’s rights activism and in activities at several mining sites in Kabare: “Even if we report certain problems, it is difficult to get help because the authorities are not always present at the mining sites”. Overall, these results highlight that the dynamics of trafficking around the mining sites of Kabare and Kalehe are part of a local system of actors characterised by multiple, ambiguous and sometimes contradictory roles. While certain economic and security practices contribute to reinforcing the structural vulnerabilities of women and girls, forms of social and institutional regulation also exist. Nevertheless, these often remain limited in the face of the rapid transformations brought about by the expansion of the artisanal mining economy and the restructuring of social relations in these extractive territories.

## Discussion

The results of this study reveal the complexity of gender dynamics in the artisanal mining sites of Kabare and Kalehe, highlighting multidimensional forms of vulnerability that profoundly affect women and girls. The analysis of these data is part of a broader body of work on artisanal mining in the Democratic Republic of Congo, which for several decades has highlighted the ambivalent nature of mines as spaces of both economic opportunity and socio-health risks (Geenen, 2014; Bashwira et al., 2014). This discussion articulates the results around three major dimensions: the ambivalent role of mines as economic spaces, the persistence of gendered power relations, and the structural implications related to governance and human security. Indeed, although artisanal mining sites play a central economic role for women and girls, often in the absence of local alternatives, they are accompanied by unequal power relations that limit their autonomy. This observation is consistent with the work of Hilson (2016), which shows that artisanal and small-scale exploitation of natural resources is, in many African contexts, the employer of last resort for marginalised populations. As we have demonstrated in this article and observed in the field, women participate in this system through a variety of activities such as crushing, washing, sorting and small-scale trading, which provide them with daily, albeit volatile, income and a degree of financial autonomy.

However, these activities are part of a cycle of structural precariousness, inspired by patriarchal logic that values the work and autonomy of men in the mining sector over other social categories such as women and girls. For example, low wages, irregular earnings and dependence on operators or miners perpetuate an unstable subsistence economy. These observations corroborate Cuvelier (2010) analysis that women working in the least profitable segments of the mining chain remain trapped in a deeply unequal extractive system. The study also highlights the growing presence of very young girls in mining activities, often to finance their education or subsistence. This dynamic reflects not only the precariousness of households, but also the absence of institutional mechanisms to guarantee children’s rights in mining areas, as also noted by Spiegel (2012), André and Godin (2014), and Ulimwengu and Sanginga (2025) in their work on critical minerals in the Democratic Republic of Congo.

Respondents emphasised that despite the precarious nature of the work, the results show that mining sites offer opportunities for women’s empowerment, as they gain access to their own income, greater mobility and a degree of fulfilment, as well as new social networks. These elements correspond to what Cornwall (2007) describes as ‘spaces of agency within oppressive structures. The opportunity for women to generate independent income can be interpreted as a form of economic empowerment, echoing Lahiri-Dutt (2011) observations on artisanal mines in Latin America, South Asia and Africa. However, this autonomy remains relative, as it develops in a male-dominated environment controlled by informal hierarchies and marked by asymmetrical power relations. Women’s solidarity networks, notably AVECs, associations of “Shahouleurs” and groups of porters, appear to be collective mechanisms of resilience. These forms of local organisation reflect what Scott (1985) calls “arts of resistance,” enabling subaltern groups to circumvent structural constraints. However, their capacity for action remains limited by the lack of institutional support, the low recognition of their economic role, and the persistence of patriarchal attitudes in mining decision-making structures (cooperatives, local committees, traditional authorities). The study highlights the persistent invisibility of women’s work in mines. This invisibility is documented in several studies on the DRC (Bashwira et al., 2014; Babo, 2025), which show that women’s work is often considered non-technical, unskilled or secondary. This representation directly influences their position in the mining value chain, confining them to low-paid tasks. The gendered division of labour is rooted in deeply entrenched patriarchal norms. The belief that the presence of women, particularly during menstruation, causes fish stocks to “disappear” echoes the symbolic representations analysed by Douglas (1998) around “purity” and “danger.” These beliefs serve as a cultural justification for the exclusion of women, reinforcing structural discrimination. The testimonies collected also highlight the existence of a continuum of symbolic, economic and sexual violence. These forms of exploitation are part of what Bourdieu (1998) describes as “male domination”, where gender relations are reproduced through invisible but effective mechanisms of inferiorisation.

The results of this study also highlighted that the dynamics of trafficking and exploitation of women and girls around the mining sites of Kabare and Kalehe are part of a local system characterised by the interaction of multiple actors whose roles appear both ambivalent and structuring. These results are consistent with several existing studies on artisanal extractive economies in Africa. Indeed, the strong presence of informal economic activities around mines fosters the emergence of an environment conducive to various forms of exploitation. This observation confirms the analyses of Arthur-Holmes et al. (2025), which shows that the informality of mining sites and the lack of effective regulation encourage the sexual exploitation of women in artisanal mines in Ghana. These conditions allow male networks to control access to women, reinforcing coercion and sexual abuse. Furthermore, the testimonies collected in this study reveal that the power dynamics around mining sites are strongly influenced by the presence of security actors and, in some cases, armed groups. The women interviewed describe an environment marked by insecurity and power relations that can force some of them to accept sexual relations in order to preserve their economic activity or personal safety. These observations are consistent with the work of Rustad et al. (2016), who emphasise that the presence of armed actors and the lack of clear supervision by local authorities increase the risk of sexual and physical violence against women. Women living near mining sites thus become particularly vulnerable to exploitation and perpetrator impunity. In artisanal mining contexts, these security dynamics reinforce the vulnerability of women who live or work near extraction sites, while promoting perpetrator impunity.

Beyond these local dynamics, the findings of this study must also be understood within broader historical and political-economic systems of power that shape extractive economies in the Global South. Scholars of political ecology and ecofeminism have argued that contemporary mining economies cannot be separated from the historical legacies of colonial extraction and the expansion of global capitalist markets, which have long positioned resource-rich regions such as Central Africa as peripheral zones of extraction for the global economy (Bridge, 2008). Within these systems, natural resources, territories and labour are incorporated into global commodity chains in ways that often reproduce structural inequalities and asymmetrical power relations. From an ecofeminist perspective, these dynamics also involve the simultaneous exploitation of nature and women’s labour and bodies, as both are frequently treated as resources to be appropriated within extractive capitalist systems (Mies et al., 2022).

Recent feminist political ecology scholarship further emphasises that extractive frontiers often become sites where environmental dispossession, economic marginalisation and gendered violence intersect (Jenkins, 2014; Lahiri-Dutt, 2011). In such contexts, the transformation of landscapes through mining is accompanied by the reconfiguration of social relations and gender hierarchies, which may intensify women’s vulnerability while simultaneously creating limited spaces for economic participation. Decolonial ecofeminist perspectives conceptualise this interconnection through the notion of cuerpo-territorio (body-territory), which views women’s bodies and territories as interconnected sites affected by extractivist violence and domination (Zaragocin and Caretta, 2021). This perspective helps to situate the experiences of women in Kalehe and Kabare within broader systems of extractivism, patriarchy and global economic structures that shape both territorial exploitation and gendered inequalities.

## Conclusion

At the end of this study, the mining sites of Kabare and Kalehe appear to be deeply ambivalent spaces for women and girls, places that are essential for economic survival, but also environments where gender inequalities, precariousness and structural violence are reproduced and intensified. Mines are not only a source of income; they have become an invisible pillar of social reproduction in rural households, relying heavily on the undervalued and unrecognised work of women. Yet women continue to occupy the most precarious segments of the mining chain, while simultaneously bearing the burden of domestic and family responsibilities. That said, while mines offer real opportunities for economic autonomy, mobility and the building of solidarity networks, these forms of emancipation remain fragile and incomplete, as they are part of an extractive system deeply marked by unequal power relations. Access to mining resources remains conditioned by patriarchal and clientelist logics that limit the ability of women and girls to transform these economic opportunities into real social and economic power.

Thus, far from being reduced to a passive position as victims, women working in mining areas appear instead as key players in social and economic resilience, negotiating their place on a daily basis in an environment marked by significant material, institutional and security constraints. Through various adaptation strategies, whether diversifying economic activities, mobilising informal solidarity networks or innovating in mineral processing and marketing activities, they actively participate in the structuring and functioning of the local mining economy. Their contribution thus goes beyond the peripheral roles with which they are often associated and is part of a broader dynamic of social reproduction and support for household livelihoods in mining areas. However, this capacity for adaptation and initiative should not obscure the power relations and structural inequalities that continue to shape their participation in the mining sector. The autonomy enjoyed by women and girls in these spaces appears to be largely restricted, shaped by limited access to resources, marginalisation in mining value chains and increased exposure to physical, social and security risks. In this sense, the resilience observed in mining sites cannot be interpreted as a sign of true emancipation, but rather as the product of an adapted capacity developed in the face of deeply unequal structural conditions.

Ultimately, this study shows that as long as women’s work remains invisible, their access to mining resources remains limited, and institutional protection mechanisms are lacking, mining sites will continue to function as paradoxical spaces where opportunities for emancipation coexist with the reproduction of vulnerabilities. Fully recognising women’s contributions, strengthening their access to resources and integrating their voices into mining governance therefore appear not only as imperatives of equity, but also as essential conditions for more inclusive and sustainable mining in the Kabare and Kalehe territories.

## Data Availability

The original contributions presented in the study are included in the article/supplementary material, further inquiries can be directed to the corresponding author.

## Footnotes

1 The term “Shahouleur” refers, in the context of mining sites in South Kivu (particularly in Kalehe and Kabare), to women who are primarily responsible for cleaning, sorting and preparing the extracted ore. They do not generally dig in the pits themselves but play an essential role in the mining value chain by making the ore usable and marketable. These women may also organise themselves into associations or collective groups to better structure their activity, negotiate the sale of minerals or sand, and support each other. The term thus reflects a specific and socially recognised economic function, even if it often remains invisible and undervalued compared to that of male miners.
